# Integrated Analysis of Volatile Metabolites in Rose Varieties: Effects of Cultivar Differences and Drying Temperatures on Flavor Profiles

**DOI:** 10.3390/metabo15050325

**Published:** 2025-05-14

**Authors:** Jun Zhang, Meile Sun, Xiangrong Ren, Jing Yang, Yijie Zhang, Jingtao Hui, Pengbing Li, Jianfei Tao, Tianzhi Liu, Guocang Lin

**Affiliations:** Xinjiang Academy of Agricultural Sciences Comprehensive Experimental Farm, Urumqi 830012, China; zj001vip@163.com (J.Z.); sunmeile@xaas.ac.cn (M.S.); renxiangrong@xaas.ac.cn (X.R.); yangjing@xaas.ac.cn (J.Y.); zhangyijie@xaas.ac.cn (Y.Z.); huijingtao@xaas.ac.cn (J.H.); lipengbing@xaas.ac.cn (P.L.); taojianfei@xaas.ac.cn (J.T.); ygsm2024@163.com (T.L.)

**Keywords:** rose flowers, hot air drying, variety, volatile substances, WGCNA

## Abstract

Background: Rose processing faces critical challenges in preserving bioactive compounds and aroma profiles during thermal treatments, particularly given the growing demand for natural ingredients in the food and cosmetic industries. Methods: Using widely targeted metabolomics, we first characterized volatile profiles of four major commercial cultivars (Hetian, Damask, Bulgarian, and Fenghua; *n* = 6 replicates per cultivar), identifying terpenoids as dominant components (*p* < 0.05). Subsequent thermal optimization focused on Hetian rose, where WGCNA and K-means analyses revealed temperature-dependent dynamics (40–55 °C, triplicate drying trials per temperature). Results: Hetian rose exhibited significantly higher accumulation (*p* < 0.05) of a unique sesquiterpene marker, 4-(1,5-dimethyl-1,4-hexadienyl)-1-methyl-cyclohexene. Systematic drying optimization identified 50 °C as the thermal threshold for optimal color, bioactive retention, and sensory quality. Mechanistic analysis identified 193 temperature-responsive metabolites (VIP > 1, FC < 0.25 or >4, *p* < 0.01), with terpenoid biosynthesis (MVA/MEP pathways) and esterification dynamics emerging as critical control points. Conclusions: This study establishes the first cultivar-specific processing framework for roses, demonstrating that metabolic signature-guided drying improves product quality. The findings advance our understanding of thermal impacts on aroma biochemistry while providing actionable protocols for natural product industries.

## 1. Introduction

Roses have served humanity for millennia as ornamental plants, medicinal resources, and culinary ingredients. Beyond their esthetic appeal, these flowers hold substantial economic value across global industries, contributing over $10 billion annually to fragrance production, natural flavorings, and herbal medicine markets [1]. This commercial significance stems from roses’ unique biochemical composition, particularly their volatile organic compounds and bioactive metabolites that determine sensory quality and therapeutic potential [1,2]. Among commercially cultivated species, four varieties stand out for their distinctive properties: Rosa damascena (*Damascus rose*), Rosa bulgarica (*Bulgarian rose*), Rosa rugosa from Fenghua (*Fenghua rose*), and the hybrid Rosa sertata × Rosa rugosa (*Hetian rose*). *Damascus* and *Bulgarian roses* dominate perfume production due to their characteristically high concentrations of citronellol (up to 42.2% in *Damascus rose* essential oil) and geraniol (37.5%)—values significantly exceeding those of other commercial cultivars [3,4]. The Fenghua rose has carved a niche in food applications, with its anthocyanin-rich, thick-petaled flowers demonstrating superior stability during high-temperature drying processes used in tea production [5]. Hetian rose emerges as a particularly versatile candidate, combining unique organoleptic properties with remarkable thermal resilience. Its linalool–eugenol aroma profile creates a distinctive sweet–spicy bouquet, while evolutionary adaptation to Xinjiang’s arid climate has endowed it with thick, waxy petals that retain 30% more volatiles during dehydration compared to other varieties [6]. These characteristics position Hetian rose as an ideal candidate for premium dried products, though its full potential remains constrained by insufficient optimization of critical processing parameters like drying temperature (40–55 °C).

The dehydration process profoundly impacts rose quality metrics—excessive heat degrades anthocyanins (color), accelerates terpene evaporation (aroma loss), and denatures heat-sensitive polyphenols [5,7,8]. While higher temperatures (60 °C) reduce drying time by 40%, they concurrently induce color fading and reduce phenolic content by 22% [9,10]. Emerging techniques like sodium isoascorbate pretreatment show promise in color preservation, yet temperature remains the primary modifiable factor [11]. Current optimization efforts focus narrowly on single parameters—for instance, 60 °C for 4.5 h improves color retention in Bulgarian roses but fails to preserve their complete volatile profile [12]. This highlights the need for variety-specific process optimization, particularly for thermally robust varieties like Hetian rose, whose biochemical complexity demands tailored approaches [13]. Therefore, we set the experimental temperature of this experiment at 40–55 °C according to the above findings.

Metabolomics has revolutionized quality analysis in plant processing by enabling comprehensive mapping of metabolic responses to environmental stresses [14,15]. Recent studies demonstrate its utility in rose research: UPLC-Q-TOF-MS analyses by Zou et al. [16] revealed flavonoid composition differences across three rose varieties, correlating specific metabolites with color phenotypes. Similarly, Kanani et al. [17] tracked stage-specific phenolic changes in R. damascena, identifying key compounds influencing harvest timing decisions. These advances underscore metabolomics’ potential to decode the biochemical foundations of rose quality attributes—information critical for optimizing processing protocols while preserving bioactive constituents.

This study addresses the following two critical gaps: (1) the lack of comparative metabolic profiles for key commercial rose varieties and (2) insufficient understanding of heat-induced metabolic changes during Hetian rose processing. We employ broad-spectrum metabolomics to systematically analyze volatile profiles in four rose varieties (Hetian, Bulgarian, Damascus, and Fenghua), identifying characteristic metabolite signatures. Building on observed thermal resilience in Hetian roses, we then optimize its drying protocol through temperature-gradient experiments (40–55 °C). By integrating metabolic pathway analysis with quality metrics, we elucidate how varietal differences and processing parameters influence key biochemical pathways governing aroma and color stability. These findings advance rose product standardization efforts while providing a framework for cultivar-specific process optimization in the flavor and cosmetics industries.

## 2. Materials and Methods

### 2.1. Materials and Reagents

Fresh flowers of *Rosa rugosa* (HT, Hetian rose), *Rosa × damascena* (DM, Damascus rose), *Rosa × damascena* (BG, Bulgarian rose), and the *hybrid Rosa sertata × Rosa rugosa* (FW, Fung Wah rose) were collected in June from Wanfang Village, Arele Township, Yutian County, Hetian Prefecture, Xinjiang Uygur Autonomous Region, China (36.81° N, 81.66° E; average altitude: 5292 m). Six biological replicates (*n* = 6) per variety combination were processed simultaneously. The chemical reagents, including ethanol, sodium nitrite, aluminum nitrate, and sodium chloride, were purchased from the China National Pharmaceutical Group Corporation (Beijing, China), while the n-hexane was obtained from Merck (Darmstadt, Germany). The analytical standards (purity > 98%) were sourced from BioBioPha and Sigma-Aldrich (Shanghai, China).

### 2.2. Methods

#### 2.2.1. Flower Drying Method

Fresh flower buds of four rose cultivars were selected based on uniform coloration, identical developmental stages, and absence of physical damage or pest infestation. Drying experiments were conducted in a programmable hot-air drying chamber (DHG-9011A, Shanghai Jinghong Experimental Equipment Co., Shanghai, China) equipped with precision temperature control (±0.5 °C), a humidity regulation system (maintained at 18 ± 2% RH), and an airflow velocity of 1.5 m/s (±0.1 m/s). For comparative metabolomic analysis, all four cultivars underwent gradient drying trials at 40 °C, 45 °C, 50 °C, and 55 °C until reaching constant mass (operationally defined as <0.1% weight variation over 2 consecutive hours, verified by continuous gravimetric monitoring) [13]. Six biological replicates (*n* = 6) per temperature–variety combination were processed simultaneously.

#### 2.2.2. Total Flavonoid Determination

Fresh Hetian rose flowers were freeze-dried to remove moisture, ground into a fine powder using a QE-200 portable pulverizer (Hangzhou Sansi Instrument Co., Hangzhou, China), and passed through an 80-mesh sieve with a 0.180 mm aperture. A 90% ethanol solution was added at a material-to-liquid ratio of 1:20 (g/mL), thoroughly mixed, and allowed to stand for 10 min. Next, the sample was subjected to ultrasonic extraction at 40 °C for 10 min, followed by centrifugation at 10,000 r/min for 5 min, after which the supernatant was collected as the sample solution for further analysis.

After sample preparation, the total flavonoid content was determined using a colorimetric method. An appropriate amount of the sample solution was transferred into a 5 mL test tube, after which 0.3 mL of a 5% sodium nitrite solution and 0.3 mL of a 10% aluminum nitrate solution were added. The mixture was vortexed thoroughly and allowed to stand for 6 min at room temperature, followed by the addition of 2 mL of a 4% sodium hydroxide solution. The volume was adjusted to the mark with distilled water, mixed thoroughly, and left to stand for an additional 10 min, after which the absorbance of the final solution was measured at 510 nm using the UV 2600 spectrophotometer (Shimadzu Corporation, Kyoto, Japan).

#### 2.2.3. Total Anthocyanins Determination

The sample was prepared as previously described for total flavonoid determination [18]. The powdered samples were extracted using 90% ethanol at a material-to-liquid ratio of 1:20 (g/mL), thoroughly mixed, and allowed to stand for 10 min, followed by ultrasonic extraction at 40 °C for 10 min and centrifugation at 10,000 r/min for 5 min. The supernatant was collected and acidified with hydrochloric acid to a final concentration of 0.1% *v/v* to stabilize anthocyanins and enhance color development. The absorbance of the acidified extract was measured at 530 nm using the UV 2600 spectrophotometer (Shimadzu Corporation, Kyoto, Japan), and the anthocyanin content was calculated according to the following standard calibration curve:

Anthocyanin content (mg/mL) = (absorbance × sample volume)/(molar extinction coefficient × path length)

#### 2.2.4. Sensory Evaluation

Ten food science students (with ≥2 years of sensory evaluation coursework) were recruited as panelists and underwent standardized training following ISO 8586:2023 [19] guidelines for sensory assessor competency. Each judge placed approximately 10 g of rose solution on a blank A4 paper and gently shook it to observe the appearance. Then, 3 g of the rose samples were mixed with 150 mL of boiling water in clean paper cups and allowed to steep for 8 min, after which the broth was poured out to assess the color, aroma, and flavors. The judges evaluated the samples using a predefined rating scale, after which an average score was calculated and used as the statistical result [18].

#### 2.2.5. Volatile Metabolomics Determination

The samples were removed from −80 °C refrigerator storage, ground using liquid nitrogen, and vortexed to mix well. Then, 1 g of each sample was placed in headspace vials, followed by the respective addition of a saturated NaCl solution and 10 μ (50 μg/mL) of the internal standard. The samples were subjected to fully automated headspace solid-phase microextraction for GC-MS analysis. The chromatographic conditions included a DB-5MS capillary column (30 m × 0.25 mm × 0.25 μm, Agilent & W Scientific, Folsom, CA, USA), high-purity (≥99.999%) helium as the carrier gas, a constant flow rate of 1.2 mL/min, an inlet temperature of 250 °C, non-split injection, and a solvent delay of 3.5 min. The initial temperature of 40 °C was maintained for 3.5 min, followed by an increase to 100 °C at 10 °C/min, 180 °C at 5 °C/min, and 280 °C at 25 °C/min, where it was held for 5 min. The MS conditions included an electron ion source (EI), an ion source temperature of 230 °C, a four-stage rod temperature of 150 °C, an MS interface temperature of 280 °C, and an electron energy of 70 eV. Selected ion detection mode (SIM) was used for qualitative and quantitative ion scanning (GB 23200.8-2016 [20]).

### 2.3. Data Analysis

The samples were statistically analyzed using SPSS 26 (IBM, Almonk, NY, USA) and visualized in Origin 2021b (OriginLab, Northampton, MA, USA), with significant differences (*p* < 0.05) denoted by superscript letters (a, b, and c) derived from post hoc Tukey’s HSD tests. For multivariate analyses, raw metabolomic data were first filtered to remove low-abundance metabolites (detected in <50% of samples). The retained data were then normalized via quantile normalization (R package preprocessCore v1.52.1) and log2-transformed to stabilize variance. Principal component analysis (PCA) was performed using the prcomp function in R (v4.2.1) with unit variance scaling (UV scaling) applied to center and scale the variables. Hierarchical clustering heatmaps were generated on ChiPlot (www.chiplot.online, accessed on 16 December 2024) using Euclidean distance as the similarity metric and Ward’s minimum variance method for dendrogram construction. K-means clustering was implemented on the Metware Cloud platform (https://cloud.metware.cn/, accessed on 20 December 2024) with the optimal cluster number (k = 6) determined by elbow method analysis (sum of squared errors vs. k; threshold at 90% variance explained). Weighted gene co-expression network analysis (WGCNA) was conducted using the R package WGCNA (v1.72) with soft thresholding power set to 12 (scale-free topology fit > 0.85) and a minimum module size of 30 metabolites. Module–trait associations were calculated using Pearson correlation with Benjamini–Hochberg FDR correction.

## 3. Results and Analysis

### 3.1. Analysis of Volatile Metabolites in Different Varieties of Fresh Flowers and After Drying

**Figure 1:**
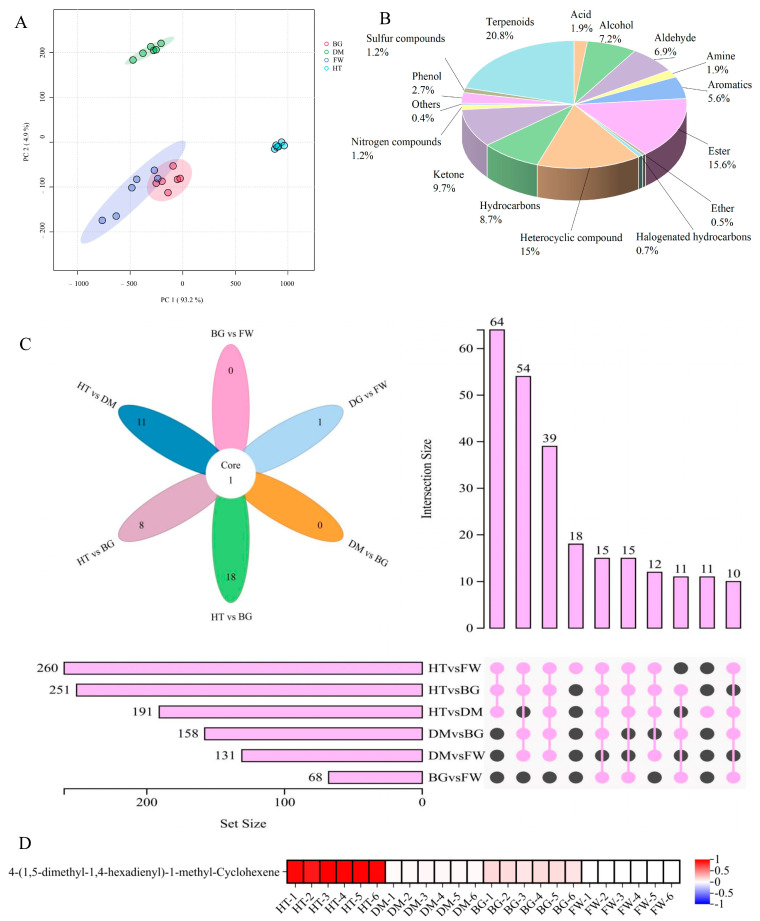
Differential analysis of volatile metabolites in fresh rose flowers. (**A**) PCA of four rose flowers (different groups are made up of different colored circles); (**B**) pie chart of substance classification in four rose flowers; (**C**) Wayne diagram of four rose flowers; and (**D**) heat map of differential metabolites, determining the amount of the substance in different treatments based on the color of the scale.

Analysis of the composition of volatile metabolites in Hetian, Damask, Bulgarian, and Fenghua roses revealed that the volatile substances in Hetian and Damask roses were significantly different from those in the remaining two roses (Figure 1), and similar volatile compositions in Bulgarian and Fenghua roses could be seen from the PCA analyses. A total of 16 classes of substances were detected in the four roses, with terpenes having the highest percentage of 20.8%.

A total of 751 volatile metabolites were detected in the four fresh roses, which were screened for differential metabolites contained in each of the four roses using the conditions of VIP > 1, *p* ≤ 0.01, and FC ≥ 4 or ≤0.25, as shown in Figure 1C. The only differential metabolite shared between the groups was 4-(1,5-dimethyl-1,4-hexadienyl)-1-methyl-cyclohexene, which is a sesquiterpenoid among terpenoids and is mainly involved in the aroma formation and defense function of roses through the terpenoid metabolic pathway in roses, and its structural features and reactivity make it a key intermediate of monoterpenes, sesquiterpenes, and derived secondary metabolites. The highest content of this substance was found in Hetian rose (Figure 1D), suggesting that Hetian rose generates unique aroma molecules through the terpenoid metabolic pathway, and 4-(1,5-dimethyl-1,4-hexadienyl)-1-methyl-cyclohexene is the signature volatile of Hetian rose (*p* < 0.01).

**Figure 2:**
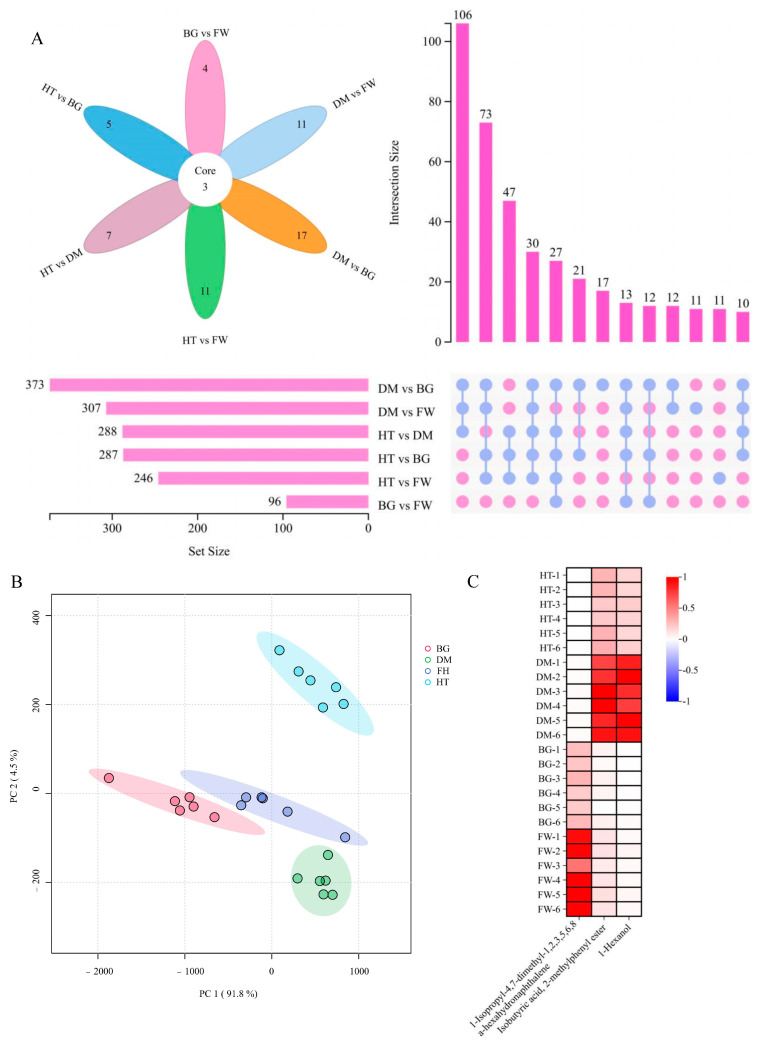
Differential analysis of volatile metabolites in dried roses. (**A**) Wayne plots of four dried roses; (**B**) PCA of four dried roses, different groups are made up of different colored circles; (**C**) heat map of differential metabolites, determining the amount of the substance in different treatments based on the color of the scale.

Dried roses were made from Hetian rose, Damascus rose, Bulgarian rose, and Fenghua rose using hot air drying at 45 °C, and the volatile composition and content of dried roses were analyzed using broadly targeted metabolomics. The volatile metabolites of dried Hetian rose among the four dried roses were significantly different from those of the remaining three dried roses (Figure 2B). The remaining three dried roses were in quadrants two and three, and the Hetian rose was in the first quadrant, which indicated that specific volatile metabolites were contained in the Hetian roses after the dry processing treatment. According to the differential metabolite screening and Wayne diagram (Figure 2A) analysis, three differential metabolites were found in the four dried roses, namely, terpene 1-isopropyl-4,7-dimethyl-1,2,3,5,6,8a-hexahydronaphthalene, ester isobutyric acid, 2-methylphenyl ester, and the alcohol 1-hexanol (Figure 2C).

There were significant varietal differences in the volatile metabolite composition of Hetian, Damask, Bulgarian, and Fontana roses, with fresh roses having terpenes (20.8%) as the core constituents, monoterpenes (e.g., geraniol) and sesquiterpenes (e.g., β-stigmastadiene) conferring fresh floral and woody finishes, respectively, and ethers (15.6%) and heterocyclic compounds (15.1%) synergistically enhancing the aroma hierarchy. The highest content of the iconic sesquiterpene 4-(1,5-dimethyl-1,4-hexadienyl)-1-methyl-cyclohexene was found in Hetian rose, suggesting that the distinctive aroma was formed through terpene metabolism. The metabolic specificity of Hetian rose was retained after drying at 45 °C, while the shared differential metabolites (e.g., sesquiterpene 1-isopropyl-4,7-dimethyl-4,7-hexadienyl-4,7-hexadienyl) were found in the rose. 7-Dimethyl-1,2,3,5,6,8a-hexahydronaphthalene, ester isobutyric acid 2-methylphenyl ester, and alcohol 1-hexanol revealed the effect of processing on the aroma: the sesquiterpene content of Hetian and Damascus roses decreased, whereas ester content increased. Moreover, the reverse trend was observed for Bulgarian and Fenghua roses. The mechanisms of temperature regulation on terpene stability, esterification/oxygenase activity, and organoleptic quality need to be analyzed through multi-gradient drying temperature experiments (40 °C, 45 °C, 50 °C, and 55 °C) to optimize the drying process and balance the retention of iconic components with the enhancement of aroma function.

### 3.2. Changes in the Color and Related Substances in the Roses in Different Drying Conditions

**Figure 3:**
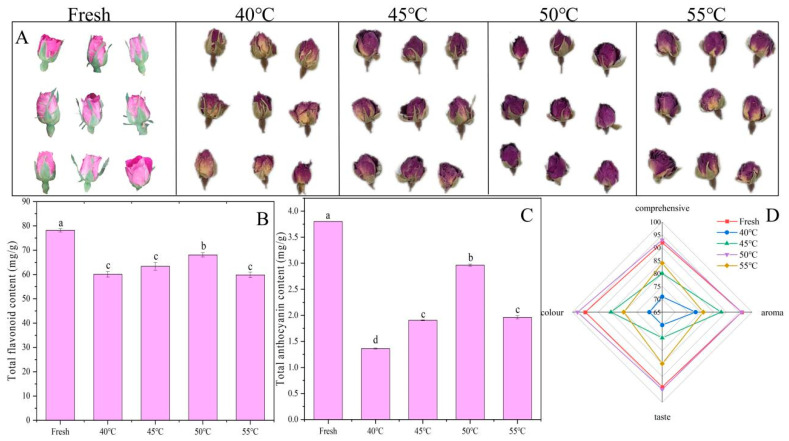
The appearance of rose samples, as well as the changes in the total flavonoid and total anthocyanin content. (**A**) The rose flower appearance and color visualization. (**B**) Total flavonoid content. (**C**) Total anthocyanin content. (**D**) A radar chart of the sensory evaluation scores of the roses. Different lowercase letters represent differentiation (*p* < 0.05).

Figure 3A,D show the sensory changes in fresh rosebuds and rosebuds dried at temperatures ranging from 40 °C to 55 °C. Colors were evaluated through the descriptions of 10 sensory evaluators, starting with the color of roses. The fresh rosebuds exhibited a pink color, which changed to varying degrees after drying. At a drying temperature of 40 °C, the roses appeared lighter in color, with a less pronounced purple hue and non-uniform coloration. At 45 °C, the samples showed initial signs of fading, with reduced brightness and increased color unevenness. Contrarily, the roses dried at 50 °C displayed a more vibrant, uniform color, with a more distinct purple tone. However, at 55 °C, the rose samples exhibited significant fading, with the purple color almost entirely disappearing and replaced by yellow and brown tones. Distinct color differences were evident among the rose groups. As the drying temperature increased, the color of the roses gradually shifted from purple to yellow and brown. As shown in Figure 3D, the drying temperature significantly influenced the sensory quality of the rose samples, which was evaluated based on four key attributes: color, taste, aroma, and overall quality. The radar diagram revealed that the roses dried at 50 °C exhibited sensory profiles similar to those of the fresh group, while other drying temperatures caused varying degrees of sensory quality degradation. The highest color score was observed at 50 °C, closely followed by the fresh group. No significant differences were evident between the aroma scores of the 50 °C and fresh groups, while the lowest value was recorded at 40 °C. Notably, drying at 40 °C resulted in the lowest sensory scores across all four attributes. These findings indicated that 50 °C was the optimal drying temperature for retaining the sensory quality of the roses.

Figure 3B illustrates the total flavonoid content in the rose samples in different drying conditions. The fresh rosebuds exhibited the highest total flavonoid content. Hot air drying at 40 °C slightly reduced the total flavonoid level (*p* < 0.05), indicating that even a relatively low drying temperature affected flavonoid stability. A drying temperature of 45 °C further decreased the total flavonoid content (*p* < 0.05), suggesting that higher temperatures exacerbated flavonoid degradation. This trend continued at 50 °C, with a more significant decline. The lowest total flavonoid level was recorded at 55 °C, demonstrating that high-temperature drying had the most pronounced destructive effect on the flavonoids. These results indicated that the hot air-drying temperature significantly influenced the total flavonoid content in the roses. Additionally, high temperatures may enhance endogenous enzyme activity, such as polyphenol oxidases, which further catalyze flavonoid oxidation.

Figure 3C illustrates the anthocyanin content variation at different drying temperatures. The fresh Hetian rose samples displayed the highest anthocyanin content, which decreased when the drying temperature was increased to 40 °C, likely due to high-temperature-induced degradation or structural changes. At 45 °C, the anthocyanin content increased significantly, reaching approximately 2 mg/g, possibly due to the stabilization or enhanced release when exposed to moderate heat treatment. The anthocyanin content exhibited a further increase at 50 °C, with levels close to 2 mg/g, suggesting that this temperature optimized anthocyanin synthesis or activated protective mechanisms. The anthocyanin level peaked at over 3 mg/g when dried at 55 °C, indicating that this temperature maximized anthocyanin synthesis or retention in Hetian roses. Alternatively, the increase in anthocyanin content at 55 °C could be due to the transformation of certain compounds during high-temperature drying.

### 3.3. Analysis of the Metabolites in the Hetian Roses in Different Drying Conditions

**Figure 4:**
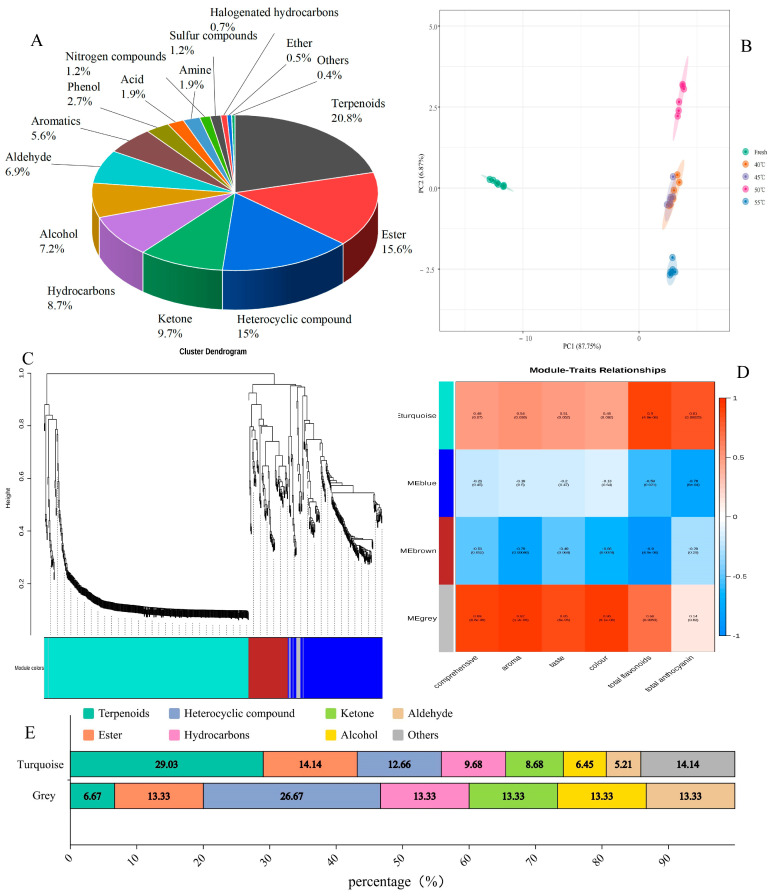
The metabolic analysis. (**A**) A metabolite classification pie chart. (**B**) A metabolite PCA plot. (**C**) Module grouping (different colors represent different module groupings). (**D**) A heatmap of the correlation between the modules and the physical and chemical quality. (**E**) A categorized stacking diagram of the substances in the module.

As illustrated in Figure 4A, the distribution of the key differential metabolites showed significant diversity in the Hetian rose samples, which was closely related to their physiological functions, aroma formation, and environmental adaptation. The terpenoid content dominated at 20.80% and was related to the plant defense mechanisms, aroma formation, and potential biological activities. The ester compounds accounted for 15.60% and were closely associated with the aromatic properties of the roses, contributing significantly to their aroma and flavor. The heterocyclic compound and ketone levels were 15.10% and 9.70%, respectively. Alcohols, aldehydes, and hydrocarbons accounted for 7.2%, 6.9%, and 8.7%, respectively, all of which were vital for the aroma and flavor of the roses. The remaining seven groups included antioxidant activity (2.7%), sulfur-containing compounds associated with specific biological activities (1.2%), acids (1.9%), amines (1.9%), trace amounts of ethers (0.5%), halogenated hydrocarbons (0.7%), and nitrogen compounds (1.2%).

The differences in the distribution and proportions of these compounds could be attributed to both the intrinsic characteristics of the rose variety and external factors such as drying temperatures. Specifically, the drying temperature and duration significantly influenced the stability and transformation of the metabolites. Furthermore, the drying temperature notably impacted the appearance and total flavonoid content of the rose tea. Lower temperatures (e.g., 40 °C) tended to preserve the color and flavonoid content, while higher temperatures (e.g., 55 °C) led to significant browning and flavonoid degradation, which aligned with the observed metabolite compositional changes.

In this study, the point clusters of all temperature-treated groups were relatively close to each other, suggesting that the drying at temperatures between 40 °C and 50 °C did not yield significant metabolic alterations compared to the fresh group. However, the 55 °C group exhibited a distinct separation from the other groups, indicating that higher drying temperatures induced more pronounced changes in the metabolic profiles. Notably, the sample distribution in the 40 °C, 45 °C, and 50 °C groups was relatively concentrated in both PC1 and PC2, highlighting a high degree of similarity among these groups. This suggests that drying at temperatures up to 50 °C preserves the metabolic integrity of the roses, while 55 °C causes significant metabolic shifts, possibly due to the degradation or transformation of heat-sensitive compounds.

These findings were consistent with the metabolic distribution analysis results (Figure 4A), further supporting the conclusion that the drying temperature played a critical role in determining the metabolic composition and overall quality of the rose samples. The distinct separation of the 55 °C group underscores the importance of optimizing drying conditions to minimize undesirable metabolic changes and preserve the bioactive properties of rose products.

The metabolic profiles of the dried roses were comprehensively analyzed using physicochemical and sensory indices. Weighted gene co-expression network analysis (WGCNA) was employed to elucidate the relationships between these indices and the metabolites. This study clustered the data into four distinct modules (Figure 4D). The turquoise and gray modules exhibited the most significant correlations with the sensory analysis outcomes and physicochemical parameters, consequently warranting further investigation of the constituent substances in these modules. The turquoise module encompassed a total of 404 substances, which were classified into eight categories. Terpenoids constituted the predominant group, followed by esters. Contrarily, the gray module comprised 15 substances, with heterocyclic compounds representing the largest proportion.

The formation of these modules is biologically significant since it highlighted metabolite groups that may co-regulate or participate in shared metabolic pathways. For instance, the predominance of terpenoids in the turquoise module suggests their potential role in influencing the aroma and sensory properties of dried roses, while the heterocyclic compounds in the gray module may contribute to specific physicochemical attributes. The WGCNA results were used for the differential screening of the 419 substances in these two modules (Figure 4E). This approach aimed to identify key metabolites that significantly contributed to the observed correlations between the sensory and physicochemical attributes in the dried roses.

### 3.4. Machine Learning-Driven Analysis of Clustering Patterns in Volatile Metabolites from Hetian Roses

**Figure 5:**
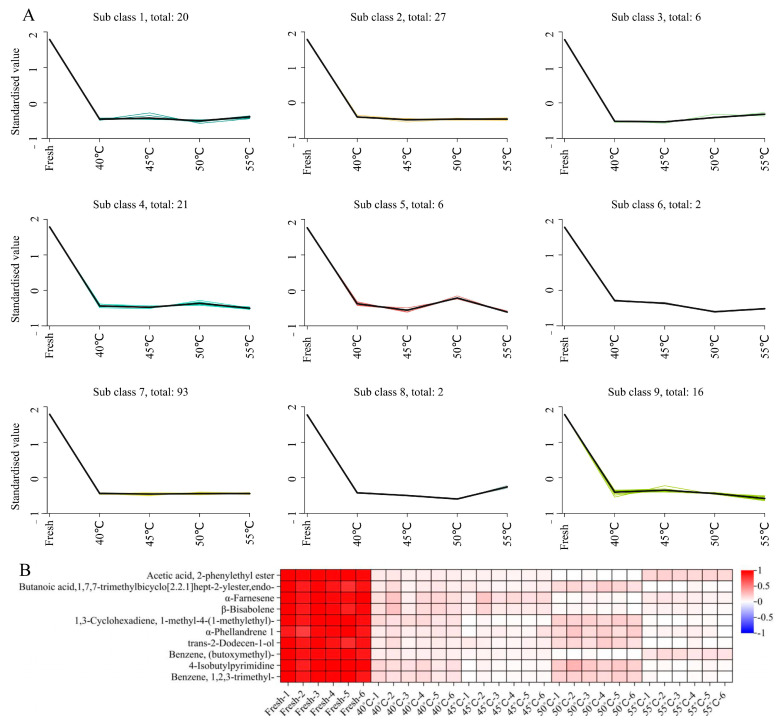
Differential metabolic analysis. (**A**) K-means analysis and (**B**) heat map of differential content.

In this study, 193 significantly different volatile metabolites were screened by WGCNA, which can be categorized into 15 classes. In order to clarify the effect of temperature on volatiles from Hetian roses, the K-means clustering algorithm was used in this study to further analyze the differential metabolites. The results showed that ten substances in subclasses 5, 6, and 8 exhibited significant responses to temperature changes (Figure 5A), while the remaining six groups remained relatively stable. These temperature-sensitive substances mainly included terpenes (4) and esters (2), and one each of alcohols, ethers, aromatics, and heterocyclic compounds.

As a straight-chain sesquiterpene, α-farnesene has a greenish, fruity, and woody aroma profile. During the drying process, its content showed a decreasing and then increasing trend (Figure 5B): the content reached the lowest point at 50 °C, and the free-state α-farnesene content briefly rebounded at 55 °C due to the rupture of plant cell membranes at high temperatures, releasing the stored farnesene pyrophosphate (FPP) precursor. β-Bisabolene showed a similar temperature response pattern, further confirming that high temperature stress on terpene metabolism. 1,3-Cyclohexadiene, 1-methyl-4-(1-methylethyl)-, and α-phellandrene 1 showed opposite temperature response trends to the other terpenes. As minor components of rose aroma, the former contributes woody tones and the latter provides a sense of coolness, and variations in the content of both may be related to different thermal stability or metabolic pathways.

### 3.5. Variety–Process Synergy Analysis Based on Metabolic Pathways

**Figure 6:**
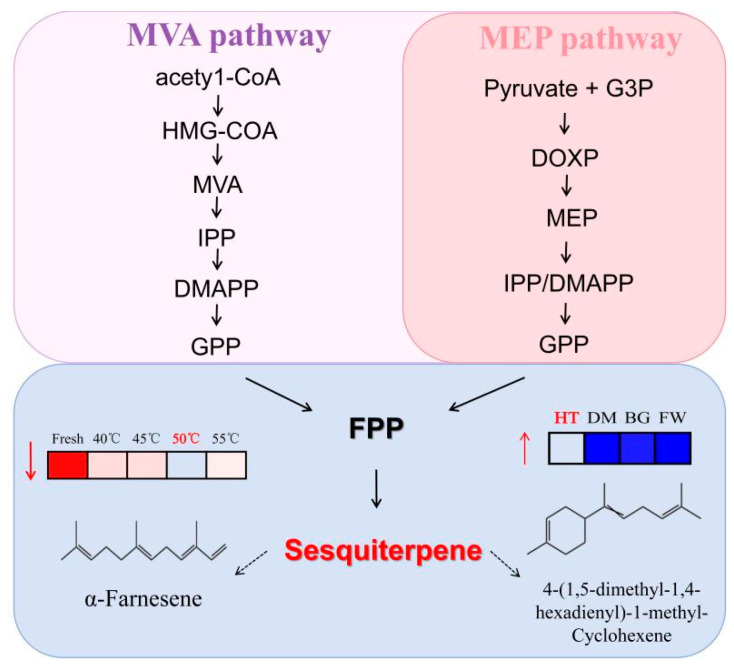
Schematic diagram of jointly analyzed metabolic pathways.

According to the results of rose variety selection and the drying process, the volatile metabolic pathway of Hetian rose is centered on terpenoids, mainly including monoterpenes (C10) and sesquiterpenes (C15), and precursors are synthesized through the mevalonate pathway (MVA) or the methyl erythritol phosphate pathway (MEP) to ultimately form the characteristic aroma of the rose (Figure 6). The distinctive aroma of Hetian rose originates from the metabolic branching of FPP for the generation of sesquiterpenes and monoterpenes, in which the reduction in α-farnesene leads to the accumulation of its precursor FPP, which may lift the feedback inhibition of the MVA/MEP pathways and promote the generation of IPP/DMAPP, which then enhances the synthesis of monoterpenes (e.g., geraniols) or other sesquiterpenes; whereas, the synthesis of 4-(1,5-dimethyl-1,4-hexadienyl)-1-methyl-cyclohexene increase competitively depletes FPP, potentially activating the upstream MVA/MEP pathways to maintain supply, but at the same time inhibiting the production of other sesquiterpenes such as α-farnesene. Changes in both together regulate the metabolic flow of FPP, thereby affecting terpene composition and aroma profiles (e.g., diminished woody tone or prominent pungent tone) in roses. This pathway provides a theoretical basis for the improvement of rose varieties and the optimization of the drying process.

## 4. Discussion

Terpenoid-driven aroma dynamics and thermal resilience. The terpenoid-dominated volatile profiles observed in the four Rosa varieties align with established phytochemical frameworks yet reveal novel structural–functional relationships. Our finding that monoterpenes (geraniol and linalool) and sesquiterpenes (β-stigmasterol) constitute the “aroma triad” through differential volatility extends the traditional view of floral scent layering. Particularly, the newly identified sesquiterpene 1-isopropyl-4,7-dimethyl-1,2,3,5,6,8a-hexahydronaphthalene in Hetian rose demonstrates exceptional thermal stability, suggesting its role as both a varietal marker and natural antioxidant. This compound belongs to the group of sesquiterpenoids, which contribute woody, resinous, or spicy aromas, enhancing complexity and persistence while synergizing with monoterpenes (e.g., linalool) to form layered fragrances [21]. The dominance of terpenoids (20.80%) aligns with their dual roles in plant defense and aroma formation [22], while their degradation at higher temperatures (e.g., 55 °C) underscores the sensitivity of terpene metabolism to thermal stress, as evidenced by β-bisabolene’s response pattern [23].

The observed color shift from purple to yellow/brown with increasing drying temperatures (40–55 °C) likely stems from anthocyanin degradation (e.g., pelargonidin-3-glucoside loss [24]) and Maillard reaction product formation (e.g., furfural accumulation [25]). Fresh rosebuds exhibited the highest flavonoid content, preserved due to the absence of thermal treatment [26]. Subsequent drying-induced flavonoid losses (e.g., 30% reduction at 50 °C) reflect accelerated glycoside oxidation into unstable aglycones and quinones [27,28]. Conversely, anthocyanins—key pigments with antioxidant and anti-inflammatory properties [29]—initially decreased at 40 °C but rebounded at 45–55 °C, suggesting heat-induced structural stabilization or precursor release.

Isobutyric acid, 2-methylphenyl ester—a fruity ester significantly enriched in dried Hetian and Damask roses—exemplifies temperature-driven esterification dynamics. This compound’s accumulation parallels the degradation of heat-sensitive terpenoids and phenolics [25], highlighting a metabolic trade-off between aroma preservation and pigment stability. Notably, 1-hexanol—an alcohol with grassy notes—serves as a precursor for aldehydes (e.g., hexanal) and esters (e.g., hexyl acetate) through oxidative pathways [30]. Its depletion at 55 °C in Damask roses challenges the conventional paradigm of sesquiterpenes as passive fixatives, instead implicating alcohol-derived volatiles in aroma modulation under thermal stress [31].

2-Phenylethyl acetate, synthesized via reversible esterification of phenylethanol and acetic acid [32,33], displayed a U-shaped thermal response: initial volatilization losses followed by precursor-driven recovery. This compound’s sweet floral aroma is critical to dried rose quality [34], while minor components like norbornyl butyrate (camphor-like notes) and trans-2-dodecen-1-ol (oxidizing to aldehydes [35]) further define cultivar-specific profiles. From an industrial perspective, the identified markers (α-farnesene and trans-2-dodecen-1-ol) enable precision drying control to balance terpenoid retention, ester enhancement, and pigment stability—key determinants of commercial viability [25].

## 5. Conclusions

This study establishes a critical linkage between rose varietal characteristics and processing parameters through systematic metabolomic profiling. The following three pivotal advances emerge from our findings: (1) Varietal Authentication Framework. The identification of 4-(1,5-dimethyl-1,4-hexadienyl)-1-methyl-cyclohexene as a potential chemical marker for Hetian rose provides an actionable tool for botanical authentication. This addresses the cosmetic industry’s urgent need for traceability in natural ingredient sourcing, particularly given the 30–40% price premium for authenticated Hetian rose derivatives in Asian markets. (2) Precision Drying Protocol. Validation of 50 °C as the thermal threshold for preserving both anthocyanin-based coloration and terpenoid-mediated aroma profiles enables standardized processing. (3) Metabolic Engineering Targets. Characterization of temperature-sensitive terpenoid nodes (α-farnesene and β-bisabolene) in the MVA/MEP pathways reveals strategic biotechnological intervention points. Future research directions should prioritize the lifecycle assessment of proposed protocols for industrial scalability. These translational insights bridge the gap between phytochemical research and commercial applications, offering a roadmap for value-added utilization of rose genetic resources in natural product industries.

## Data Availability

The data for this study are available upon request from the corresponding author.

## References

[1] Zang F., Yan M., Wu Q., Tu X., Xie X., Huang P., Tong B., Zheng Y., Zang D. (2023). Resequencing of *Rosa rugosa* accessions revealed the history of population dynamics, breed origin, and domestication pathways. BMC Plant Biol..

[2] Andersson U., Berger K., Högberg A., Landin-Olsson M., Holm C. (2011). Effects of rose hip intake on risk markers of type 2 diabetes and cardiovascular disease: A randomized, double-blind, cross-over investigation in obese persons. Eur. J. Clin. Nutr..

[3] Jaydipsinh B.R., Mahendrasinh T.K., Divya V. (2024). Drying characteristics of rose flowers. J. Agric. Eng..

[4] Ahmad M.S., Mohammed A.A., Khizar H., Fohad M.H., Shaista A., Abdulhakeem A., Asdaf A., Heba K.A., Syed R.A. (2024). Different drying techniques effect on the bioactive properties of rose petals. J. King Saud Univ. Sci..

[5] Selvi K.Ç., Kabutey A., Gürdil G.A.K., Herak D., Kurhan Ş., Klouček P. (2020). The effect of infrared drying on color, projected area, drying time, and total phenolic content of rose (*Rose electron*) petals. Plants.

[6] Titisari J., Amy A. (2022). Chemical properties of red rose (*Rosa indica* L.) herbal tea with variations of temperature and drying time chemical. Jurnal Teknik Kimia.

[7] Dilta B.S., Tushar B.B., Gupta Y.C., Rajesh B., Sharma B.P. (2011). Effect of embedding media, temperature and durations on hot air oven drying of Rose (*Rosa hybrida* L.) cv. ‘First Red’. Indian J. Appl. Res..

[8] Hnin K.K., Zhang M., Wang B., Devahastin S. (2019). Different drying methods effect on quality attributes of restructured rose powder-yam snack chips. Food Biosci..

[9] Liu Z., Liu L., Han Q., Dong G.Z., Wang B., Zhang J., Lei S.M., Liu Y.G. (2023). Quality assessment of rose tea with different drying methods based on physicochemical properties, HS–SPME–GC–MS, and GC–IMS. J. Food Sci..

[10] Matłok N., Lachowicz S., Gorzelany J., Balawejder M. (2020). Influence of Drying Method on Some Bioactive Compounds and the Composition of Volatile Components in Dried Pink Rock Rose (*Cistus creticus* L.). Molecules.

[11] Bao T., Karim N., Mo J., Chen W. (2023). Ultrasound-assisted ascorbic acid solution pretreated hot-air drying improves drying characteristics and quality of jujube slices. J. Sci. Food Agric..

[12] Zhao Y., Chong Y., Hou Z.-H. (2023). Effect of different drying techniques on rose (*Rosa rugosa* cv. *Plena*) proteome based on label-free quantitative proteomics. Heliyon.

[13] Qu F., Zhu X., Ai Z., Ai Y., Qiu F., Ni D. (2019). Effect of different drying methods on the sensory quality and chemical components of black tea. LWT.

[14] Wang S., Du Z., Yang X., Wang L., Xia K., Chen Z. (2022). An Integrated Analysis of Metabolomics and Transcriptomics Reveals Significant Differences in Floral Scents and Related Gene Expression between Two Varieties of *Dendrobium loddigesii*. Appl. Sci..

[15] Zhao J., Song Z., Joshi V.S., Khan I.A. (2010). Metabolomics Approach for Understanding the Processing of Honeysuckle Flower (*Lonicera japonica*) in Traditional Chinese. Med. Planta Medica.

[16] Zou H., Zhou L., Han L., Lv J., Jia Y., Wang Y. (2022). Transcriptome profiling reveals the roles of pigment formation mechanisms in yellow Paeonia delavayi flowers. Mol. Genet. Genom..

[17] Mehran K., Esmaeil C., Ali A.S., Mousa T.G. (2021). Plant secondary metabolism and flower color changes in damask rose at different flowering development stages. Acta Physiol. Plant..

[18] Zhou X., Wu Q., Wang X., Wei H., Zhang H., Wang Y. (2024). Integrative analysis of transcriptome and target metabolites uncovering flavonoid biosynthesis regulation of changing petal colors in Nymphaea ‘Feitian 2’. BMC Plant Biol.

[19] (2023). Sensory Analysis. General Guidelines for the Selection, Training and Monitoring of Selected Assessors and Specialized Sensory Assessors.

[20] (2016). Determination of 500 Pesticide and Related Chemical Residues in Fruits and Vegetables by Gas Chromatography-Mass Spectrometry.

[21] Nickolay T.T., Michele G. (2021). Principal component analysis (PCA). Multivariate Data Analysis on Matrix Manifolds (with Manopt).

[22] Jin J., Mi J.K., Savitha D., Jessica G.T., Yin J.L., Wong L., Rajani S., Chua N.H., Cheol J. (2015). The floral transcriptome of ylang ylang (*Cananga odorata* var. *fruticosa*) uncovers biosynthetic pathways for volatile organic compounds and a multifunctional and novel sesquiterpene synthase. J. Exp. Bot..

[23] Li M., Li J., Zhang R., Lin Y., Xiong A., Tan G., Luo Y., Zhang Y., Chen Q., Wang Y. (2022). Combined Analysis of the Metabolome and Transcriptome to Explore Heat Stress Responses and Adaptation Mechanisms in Celery (*Apium graveolens* L.). Int. J. Mol. Sci..

[24] Antonova D.V., Medarska Y.N., Stoyanova A.S., Nenov N.S., Slavov A.M., Antonov L.M. (2020). Chemical profile and sensory evaluation of Bulgarian rose (*Rosa damascena* Mill.) aroma products, isolated by different techniques. J. Essent. Oil Res..

[25] Faroogh S., Zahra R.G., Abolfazl A.Y., Mohammad K. (2023). Infrared and hot drying of saffron petal (*Crocus sativus* L.): Effect on drying, energy, color, and rehydration. J. Food Process Eng..

[26] Tatsuzawa F. (2020). Flower colors and flavonoids in the cultivars of *Lobelia erinus* L. (Campanulaceae). Dye. Pigment..

[27] Zhan S., Han X., Wang G., Qiu J., Zhou L., Chen S., Fang W., Chen F., Jiang J. (2022). Transcriptome analysis reveals chrysanthemum flower discoloration under high-temperature stress. Front. Plant Sci..

[28] Zhang X., Li L., He Y., Lang Z., Zhao Y., Han T., Li Q., Hong G. (2023). The CsHSFA-CsJAZ6 module-mediated high temperature regulates flavonoid metabolism in *Camellia sinensis*. Plant Cell Environ..

[29] Winda N. (2019). Anthocyanin as natural colorant: A review. Food Sci. J..

[30] Mahbuba K., Nuhu M., Mahci A.B., Manal A.M., Ronok Z., Shahnaj P., Most A.A. (2024). Evaluation of anti-inflammatory potential and GC-MS profiling of leaf extracts from *Clerodendrum infortunatum* L. J. Ethnopharmacol..

[31] Api A.M., Belsito D., Biserta S., Botelho D., Bruze M., Burton G.A., Buschmann J., Cancellieri M.A., Dagli M.L., Date M. (2019). RIFM fragrance ingredient safety assessment, benzaldehyde, CAS Registry Number 100-52-7. Food Chem. Toxicol..

[32] YoSup P., Han C.L., Kim Y.K., Kang S.S., Kang S.H., Byulhana L. (2020). Quality characteristics and antioxidant activites of ‘Chuwhangbae’ (*P. pyrifolia* Nakai) dried with different methods. Food Sci. Preserv..

[33] Li C., Ké L., Huitai C., Zongjun L. (2023). Reviewing the Source, Physiological Characteristics, and Aroma Production Mechanisms of Aroma-Producing Yeasts. Foods.

[34] Ahmed I.F., Mohammed H.A., Aftab A., Mohammad A.S., Ahmed E., Hasan S.Y. (2021). Evaluation of the composition and in vitro antimicrobial, antioxidant, and anti-inflammatory activities of Cilantro (*Coriandrum sativum* L. leaves) cultivated in Saudi Arabia (Al-Kharj) Saudi. J. Biol. Sci..

[35] Api A., Belsito D., Botelho D., Bruze M., Burton G., Cancellieri M., Chon H., Dagli M., Date M., Dekant W. (2022). RIFM fragrance ingredient safety assessment, phenylacetic acid, CAS Registry Number 103-82-2. Food Chem. Toxicol..

